# Exploring the Apoptotic-Induced Biochemical Mechanism of Traditional Thai Herb (Kerra™) Extract in HCT116 Cells Using a Label-Free Proteomics Approach

**DOI:** 10.3390/medicina59081376

**Published:** 2023-07-27

**Authors:** Jeeraprapa Siriwaseree, Yodying Yingchutrakul, Pawitrabhorn Samutrtai, Chanat Aonbangkhen, Pussadee Srathong, Sucheewin Krobthong, Kiattawee Choowongkomon

**Affiliations:** 1 Department of Biochemistry, Faculty of Science, Kasetsart University, Bangkok 10900, Thailand; jeeraprapa_sr@hotmail.com; 2 National Center for Genetic Engineering and Biotechnology, NSTDA, Pathum Thani 12120, Thailand; yodying.yin@biotec.or.th; 3 Department of Pharmaceutical Sciences, Faculty of Pharmacy, Chiang Mai University, Chiang Mai 50200, Thailand; pawitrabhorn.s@cmu.ac.th; 4 Center of Excellence in Natural Products Chemistry (CENP), Department of Chemistry Faculty of Science, Chulalongkorn University, Bangkok 10330, Thailand; chanat.a@chula.ac.th; 5 Faculty of Nursing, Praboromarajchanok Institute, Nonthaburi 11000, Thailand; pussadee@pckpb.ac.th; 6 Interdisciplinary Graduate Program in Genetic Engineering, Kasetsart University, Bangkok 10900, Thailand

**Keywords:** traditional herbs, LC-MS/MS, colorectal cancer, caspase-8, caspase-9, CDKN1A, MYC

## Abstract

*Background and Objectives:* Natural products have proven to be a valuable source for the discovery of new candidate drugs for cancer treatment. This study aims to investigate the potential therapeutic effects of “Kerra™”, a natural extract derived from a mixture of nine medicinal plants mentioned in the ancient Thai scripture named the *Takxila Scripture*, on HCT116 cells. *Materials and Methods:* In this study, the effect of the Kerra™ extract on cancer cells was assessed through cell viability assays. Apoptotic activity was evaluated by examining the apoptosis characteristic features. A proteomics analysis was conducted to identify proteins and pathways associated with the extract’s mechanism of action. The expression levels of apoptotic protein markers were measured to validate the extract’s efficacy. *Results:* The Kerra™ extract demonstrated a dose-dependent inhibitory effect on the cells, with higher concentrations leading to decreased cell viability. Treatment with the extract for 72 h induced characteristic features of early and late apoptosis, as well as cell death. An LC-MS/MS analysis identified a total of 3406 proteins. The pathway analysis revealed that the Kerra™ extract stimulated apoptosis and cell death in colorectal cancer cell lines and suppressed cell proliferation in adenocarcinoma cell lines through the EIF2 signaling pathway. Upstream regulatory proteins, including cyclin-dependent kinase inhibitor 1A (CDKN1A) and MYC proto-oncogene, bHLH transcription factor (MYC), were identified. The expressions of caspase-8 and caspase-9 were significantly elevated by the Kerra™ extract compared to the chemotherapy drug Doxorubicin (Dox). *Conclusions:* These findings provide strong evidence for the ability of the Kerra™ extract to induce apoptosis in HCT116 colon cancer cells. The extract’s efficacy was demonstrated by its dose-dependent inhibitory effect, induction of apoptotic activity, and modulation of key proteins involved in cell death and proliferation pathways. This study highlights the potential of Kerra™ as a promising therapeutic agent in cancer treatment.

## 1. Introduction

The utilization of natural products for cancer treatment has gained prominence in recent years. Natural products have proven to be a valuable source for the discovery of new candidate drugs for cancer treatment. For example, Curcumin, a compound found in rhizomes of *Curcuma longa* (turmeric), has been shown to have antiproliferative and proapoptotic effects on various cancer cells including colon cancers, prostate cancers, and lung cancers [1,2,3]. Epigallocatechin gallate, a compound found in green tea, has been shown to inhibit the growth of various types of cancer cells such as ovarian cancers and head and neck cancer [4,5]. Interestingly, a combination of Curcumin and epigallocatechin gallate exhibited potential anti-cancer activity by inducing apoptosis in various cancers [6]. Researchers are exploring compounds derived from natural products as potential alternatives for cancer treatment. One significant advantage of using crude natural products is their ability to target multiple pathways within cancer cells simultaneously. Cancer cells often activate multiple survival pathways, and natural products with their complex phytochemicals can effectively target these pathways, surpassing the effectiveness of single compounds that only focus on specific protein markers. Natural products typically contain various phytochemicals that can work together synergistically, resulting in a more potent therapeutic effect. This synergy adds to their potential as valuable treatments. In addition, utilizing crude herbal products for alternative cancer treatment offers several benefits, such as the ability to target multiple pathways, cost effectiveness, and the combined effects of different compounds working together. These factors emphasize the importance of natural products in developing new and effective cancer treatments.

“Takxila”, with the commercial name of “Kerra™” from the ancient Thai scripture named the *Takxila Scripture*, is a nine-ingredient mix of medicinal plants such as *Pterocarpus santalinus*, *Santalum album*, *Momordica cochinchinensis*, *Citrus aurantiifolia*, *Dregea volubilis*, etc. [7]. Each of these plants mentioned in the scripture has demonstrated significant potential in cancer therapeutics [8,9,10,11,12]. In addition, Kerra™ can inhibit inflammatory response and two enzymes in severe acute respiratory syndrome coronavirus 2 including the main protease and RNA-dependent RNA polymerase [7]. However, while the mixture of these medicinal plants adheres to the “Takxila” formula for alternative cancer treatment, there remains a big knowledge gap in understanding its efficacy. In cancer therapeutic aspects, the natural process of cell death known as apoptosis is usually altered in several signaling pathways [13]. Therefore, the discovery of new traditional herbs with apoptotic activity can be an effective approach for treating cancer.

Apoptosis, or programmed cell death, plays a crucial role in the development and maintenance of tissue and organ health in multicellular organisms [14]. Caspases play a crucial role as key regulators of apoptosis and are part of the cysteine endo-protease family that mediates cell death and inflammation. In mammals, caspases have been classified based on their well-defined biological functions in apoptosis, with caspase-3, -6, -7, -8, and -9. The biological process of caspase-mediated apoptosis involves two primary signaling pathways: intrinsic and extrinsic. Caspase-8 mediates the extrinsic pathway, while caspase-9 initiates the intrinsic pathway. Additionally, caspase-8 and -9 exert regulatory roles by activating downstream effector caspases, such as caspase-3, -6, or -7, which are responsible for stimulating various cellular apoptotic responses [15]. The apoptosis process helps to maintain a balance between cell division and cell death by removing damaged or abnormal cells. In the context of cancer, apoptosis is an important consideration for therapeutic strategies [13]. Cancer cells are characterized by uncontrolled cell division and the evasion of normal cell death mechanisms, which results in the growth of tumors. As a result, inducing apoptosis in cancer cells has been explored as a therapeutic approach. Generally, chemotherapy by using Doxorubicin (Dox) and radiation therapies often aim to induce apoptosis in cancer cells by causing DNA damage and triggering intrinsic apoptotic pathways or by targeting specific pathways that regulate apoptosis [16,17]. However, it is important to note that resistance to apoptosis-inducing treatments can develop over time in cancer cells, reducing their efficacy. Additionally, non-cancerous cells or normal cells can also be affected, leading to adverse effects. Hence, traditional medicine utilizing various herbal remedies with potential apoptotic activity may provide an opportunity for an alternative cancer treatment. 

Proteomics analysis is a powerful tool that allows the comprehensive analysis of the entire protein complement of cells. To clarify the beneficial and adverse effects of certain extracts, proteomics experiments are strongly required to evaluate their impact on cell lines or animal models. Proteomics has found extensive applications in studying cellular responses and biochemical pathways concerning various natural products, such as diterpenoids from *Rabdosia rubrescens*, sesquiterpenoids from *Curcuma aromatica*, and iridoid glycosides from *Gardenia jasminoides* [18,19,20]. By analyzing the changes in protein expression, proteomics can provide a detailed understanding of the molecular mechanisms underlying the effect of external stimulants on cellular processes such as apoptosis [21,22]. In addition, a proteomics analysis can be combined with other techniques, such as transcriptomics, metabolomics, and lipidomics, to provide a more complete picture of the molecular mechanisms underlying the effect of the external stimulants on cellular processes.

## 2. Materials and Methods

### 2.1. Kerra™ Extract Preparation and Cell Cytotoxicity Evaluation

The Kerra™ was extracted by shaking the capsule powder in 95% ethanol at a ratio of 1:100 (*w*/*v*) for 24 h at 37 °C. The extract was filtered with paper filter (Whatman, No. 41, pore size 20–25 µm) and concentrated at 40 °C by a rotary evaporator (Buchi rotavapor R-210, BÜCHI Labortechnik, Flawil, Switzerland). Finally, the concentrated extract was dissolved in 100% DMSO (Merck KGaA, Darmstadt, Germany). The human colorectal carcinoma cell line HCT116 was bought from the American Type Culture Collection (ATCC; CCL-247). The cells were cultured in the complete growth media of modified McCoy’s 5A medium (Gibco, Thermo Fisher Scientific Inc., Waltham, MA, USA) with 10% *v*/*v* fetal bovine serum (Gibco) and 1% *v*/*v* Antibiotic Antimycotic solution (100 units penicillin, 0.1 mg streptomycin, and 0.25 μg/mL amphotericin B) (Gibco). Cultured cell lines were incubated at 37 °C and 5% CO_2_. Cell cytotoxicity was determined based on MTT assay by the mitochondria dehydrogenase enzyme and cofactor reduction activity with 3-[4, 5-Dimethylthiazol-2-yl]-2,5-Diphenyltetrazolium Bromide (MTT; Merck KGaA, Germany) forming purple color crystals of formazan. HCT116 was seeded at a density of 1 × 10^4^ cells/well or 1 × 10^5^ cells/mL in a 96-well plate and left in an incubator for 24 h for cell adherence. The cells were treated with samples for 72 h in nine concentrations of a two-fold serial dilution of Kerra™ extract (5–0.020 mg/mL). The 0.1% DMSO was used as the control condition. Cell cytotoxicity was assessed by measuring the absorbance at 570 nm and calculating the percentage of the cell survival rate from the following calculation [23]:

Cell viability (%) = Mean OD_sample_/Mean OD_blank_ × 100

The experiments were conducted in three replications (n = 3). The 50% cell proliferation inhibitory concentration (IC_50_) was analyzed on a nonlinear regression dialog using GraphPad Prism 8 software (GraphPad Software Inc., San Diego, CA, USA). The resulting graph was presented in mean ± standard deviation (SD) of cell viability compared with the control.

### 2.2. Investigation of Apoptotic Events in HCT116 Cells

The detection of apoptosis in HCT116 cells was conducted using the Muse™ Annexin V & Dead Cell Kit (MCH100105, EMD Millipore Co., Burlington, MA, USA) following the manufacturer’s guidelines [24]. Briefly, the cells were seeded in a 6-well plate at a density of 300,000 cells per well and allowed to incubate overnight at 37 °C. The experiments consisted of three experimental groups including negative control (0.1% DMSO), positive control (0.1 μM Dox), and Kerra™ extract (73 μg/mL). The cells were incubated in a 5% CO_2_ incubator with 95% humidity for 72 h. The treated cells were harvested and trypsinized and the commercial kit protocol was applied. The treated cells were harvested using trypsinization and resuspended in a fresh culture medium. The cells were then stained with the Muse^®^ Annexin V & Dead Cell Kit (Luminex Corp., Austin, TX, USA) and incubated in the dark at room temperature for 20 min. Fluorescence intensity was measured using flow cytometry with the Muse™ Cell Analyzer (Merck, Germany). The stained cells were categorized into four groups: live cells (annexin V-/7-AAD-), early apoptotic cells (annexin V+/7-AAD-), late apoptotic cells (annexin V+/7-AAD+), and necrotic cells (annexin V-/7-AAD+). The apoptotic values were expressed as percentages of healthy, apoptotic, and dead cells in the negative control, positive control, and Kerra™ extract conditions. 

### 2.3. Sample Preparation for Label-Free Proteomics Analysis

The treated HCT116 cells were prepared for proteomics using a previously published protocol with minor modifications [25,26]. Briefly, the cells were lysed on ice using probe tip sonication at a frequency of 20 kHz and 80% amplitude for 2 s on and 3 s off for a total of 15 s in 200 μL of a lysis buffer (0.2% TritonX-100, 2 mM TCEP, 5 mM sodium chloride, and 10 mM HEPES-KOH, pH 8.0) with a protease inhibitor cocktail. The protein solution was collected by centrifugation at 15,000× *g* for 30 min and subjected to ice-cold 15% TCA/acetone precipitation (1:5 *v*/*v*) for 16 h. After precipitation, the pallet protein was reconstituted in 0.5% RapiGest SF (Waters, Wilmslow, UK) and 5 mM NaCl in 5 mM ammonium bicarbonate. A total of 40 µg of protein was subjected to gel-free-based digestion. Sulfhydryl bonds were reduced by using 1 mM TCEP in 5 mM ammonium bicarbonate at 56 °C for 1 h and alkylation of sulfhydryl groups by using 4 mM IAA in 5 mM ammonium bicarbonate at room temperature for 40 min in the dark. The solution was cleaned up with a desalting column (Zeba™ Spin Desalting Columns, 7K MWCO, 0.5 mL, ThermoFisher, Waltham, MA, USA). The flow-through solution was enzymatically digested by Trypsin (Promega, Walldorf, Germany) at a ratio of 1:40 (enzyme: protein) and incubated at 37 °C for 6 h. The tryptic peptides were dried and stored at −20 °C until LC-MS/MS analysis. 

### 2.4. LC-MS/MS Configurations for Proteomics Analysis

The proteomics analysis was performed using a high-resolution SciEx 6600+ TripleTOF system (AB-Sciex, Concord, Montreal, QC, Canada) coupled with a nanoLC system, the UltiMate 3000 LC System (Thermo Fisher Scientific, Madison, WI, USA), following previous publications with minor modifications [25]. Briefly, the dried tryptic peptides were reconstituted with 0.1% formic acid and 1.2 μg of protonated peptides was subjected to the nanoLC system. The mobile phases consisted of (A) 0.1% formic acid in water and (B) 95% acetonitrile with 0.1% formic acid. The samples were directly loaded onto a C18-reverse phase column (2 mm, 75 μm × 15 cm) and separated over a 155 min period at a constant flow rate of 300 nL/min. The mass spectrum was acquired in data-dependent acquisition mode, with full scans over a mass range of 400–1600 *m*/*z*. The top 30 most abundant peptide ions with charge states ranging from 2 to 5 were selected for fragmentation. The dynamic exclusion duration was set at 18 s. The raw MS files were annotated with referenced protein sequences using the Paragon algorithm by ProteinPilot software [27]. The reviewed database used for the Paragon algorithm was assembled in FASTA format and retrieved from UniprotKB (https://www.uniport.org) on 21 October 2022 (species: *Homo sapiens*). 

### 2.5. Protein Data and Pathway Enrichment Analysis 

To reduce the variability in the protein dataset, the normalization of protein intensity was performed using the NormalyzerDE [28], with quantile normalization applied to the relative expression data analysis after adding “1” to all expression values. To ensure high-confidence data, only proteins identified with an FDR ≤ 1% and ≥10 peptides/protein were considered for the confidential protein list. The differentially expressed proteins were shown in a volcano plot using a negative natural log of the *p*-values plotted against the base2 log values of the change in each protein between the Kerra™ extract (n = 3) and negative control group (n = 3). The effect of the Kerra™ extract on the pathway signaling cascade in HCT116 cells was analyzed using Ingenuity Pathway Analysis (IPA). All differentially expressed proteins were imported for the core analysis and analyzed to define the significantly changed protein signaling pathways and upstream regulators. The detailed procedures for IPA and its parameters are described in a previous report [29]. The analysis was performed by comparing all changed proteins against known canonical pathways within the IPA database (accessed on 18 May 2023). The activation and deactivation state of pathways and upstream regulators (any protein that can affect the expression of another protein) were analyzed based on the all differentially expressed proteins and adj. *p*-value (*z*-score). Major signal transduction pathways were reconstructed according to IPA results. Acceptable upstream regulators were required to have a *z*-score ≥ 1.5 and a *p*-value < 0.01. 

### 2.6. Immuno-Based Early Apoptosis Protein Quantification

The level of apoptotic protein markers was measured using the MILLIPLEX^®^ early apoptosis magnetic bead kit (48–669 MAG). The levels of active caspase-8 (Asp384) and active caspase-9 (Asp315) were quantitated based on Luminex^®^ xMAP^®^ technology. Different passages of HCT116 cells were used in these experiments to confirm apoptotic events in the cells. The cells were cultured according to the specified protocols. Following treatment with the Kerra™ extract at IC_50_ for 72 h, the cells were washed with ice-cold buffered saline and disrupted with 0.3 mL of 1X MILLIPLEX^®^ Lysis Buffer containing a protease inhibitor cocktail. To obtain lysed cells, the reaction was incubated at 50 °C for 10 min with manual mixing. The supernatant was collected by centrifugation at 14,000× *g* at 16 °C for 30 min. The protein concentration was measured using the BCA protein assay and adjusted with PBS to a concentration of 2 μg/μL. Prior to the experiment, the protein solution was further diluted in PBS at a 1:4 (*v*/*v*) ratio, resulting in a final concentration of 0.5 μg/μL. A total of 20 μL (10 μg) of the protein solution was subjected to the assay. For the magnetic beads, biotin-labeled detection antibody, streptavidin-PE, normalizing control proteins, and MILLIPLEX^®^ cell lysates were prepared according to the manufacturer’s instructions without any modifications. The efficiency and accuracy of immune-based reactions were qualified before the experiment. A549 cells stimulated with 5 µM camptothecin cell lysate were used as a positive control to confirm the expression profile of these apoptotic proteins, while HeLa cells treated with lambda phosphatase served as the negative control (no apoptotic characteristic cells) [30]. The quantification of protein levels was reported as the median fluorescence intensity (MFI) value along with the standard deviation based on two biological replicate experiments and two replicate wells.

### 2.7. Statistical Analysis

For the pairwise comparisons in the proteomics analysis, a protein-level one-way analysis of variance (one-way ANOVA) with two multiple testing correction methods, the Bonferroni correction and the Benjamini and Hochberg FDR correction, was performed by ProteinPilot™ software. For pathway analysis, a right-tailed Fisher’s exact test was used to calculate the significance of pathways and upstream regulators [31]. 

## 3. Results

### 3.1. Cell Cytotoxicity

**Figure 1:**
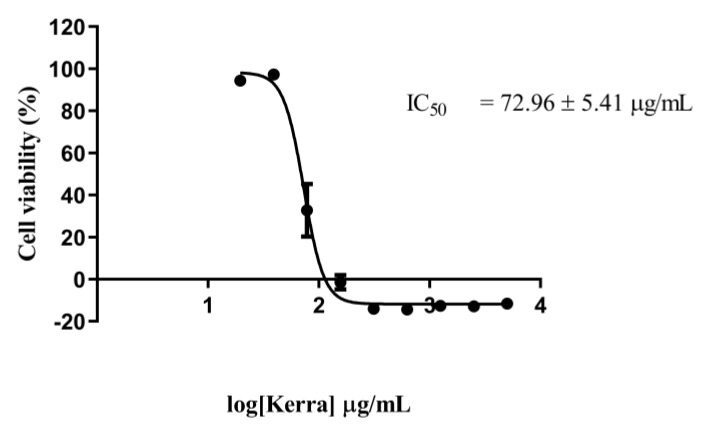
Cytotoxicity effect of Kerra™ extract against HCT116 cells after 72 h of exposure using MTT assay at the concentration ranging from 5 to 0.020 mg/mL in logarithmic scale.

The cytotoxicity effect of the Kerra™ extraction demonstrated its anti-cancer properties. The extract exhibited a dose-dependent inhibitory effect as the concentration increased. After being treated for 72 h, the viability of the HCT116 cells had slightly decreased at a concentration of 39.06 µg/mL and was completely inhibited from 156.25 to 5000 µg/mL (Figure 1). 

The IC_50_ value of the Kerra™ extract was determined to be 72.96 ± 5.41 µg/mL. In comparison, the positive control, Dox, exhibited an IC_50_ value of 0.059 µg/mL. These finding clearly indicate that the Kerra™ extract significantly affected the proliferation of HCT116 cells.

### 3.2. Kerra™ Extract Promotes Apoptosis in HCT116 Cells

**Figure 2:**
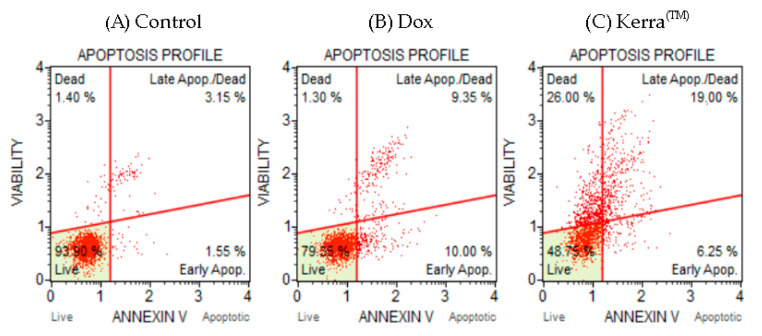
Apoptosis cell characteristic analysis using Muse™ Annexin V assay. Two-dimensional diagram of viability and Annexin V-position cells in negative control (**A**), positive control (**B**), and Kerra™ extract (**C**) groups.

Flow cytometry was used to determine the apoptotic potential of the Kerra™ extract, allowing the identification of healthy, early apoptotic, late apoptotic, and death cells. To investigate the apoptotic effect, the cells were treated with the IC_50_ concentration of the Kerra™ extract (73 μg/mL). The cell population profiles after treatments with the negative control (0.1% DMSO), positive control (0.059 μM Dox), and Kerra™ extract (73 μg/mL) are shown in Figure 2A, 2B, and 2C, respectively. 

Cells treated with the Kerra™ extract for 72 h showed early apoptosis, late apoptosis, and cell death characteristic. When the cells treated with the Kerra™ extract were compared with negative control cells, there was a significant difference in the percentage of healthy and apoptotic cells (*p*-value < 0.01). The results showed that the Kerra™ extract increased total apoptosis by 21.55% compared to the control. For confirmation, the experimental assay was corrected and the positive control was showed to increase the number of apoptotic cells in comparison to the negative control. These findings imply that the Kerra™ extract at 73 μg/mL can cause an increase in early and late apoptotic events in comparison to negative control cells (0.1% DMSO) in HCT116.

### 3.3. Comparative Proteomics Analysis 

**Figure 3:**
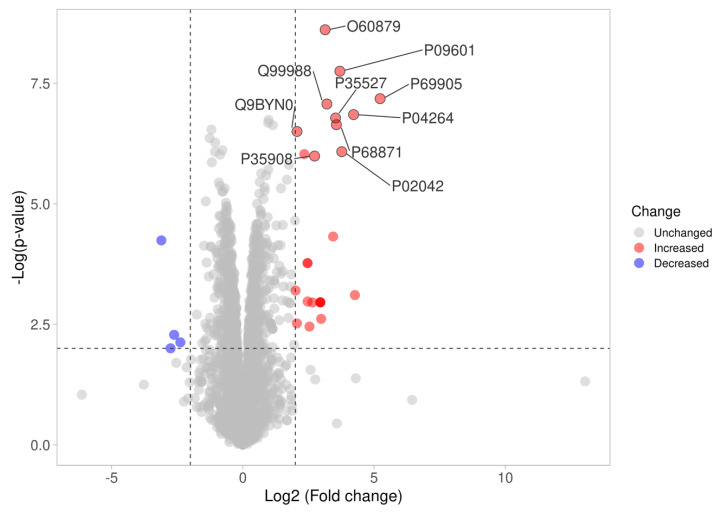
Differences in proteome expression by a volcano plot. The plot shows a negative natural log of the *p*-values plotted against the base2 log values of the change in each protein between the Kerra extract and control group (Figure 3). Significantly differentially expressed proteins were chosen by *p* < 0.01 and log2 fold change >2. The upregulated and downregulated proteins are marked as red and blue dots, respectively.

In the LC-MS/MS-based proteomics analysis of the control and treatment conditions (IC_50_ of Kerra™ extract), a total of 18,448 unique peptides corresponding to 3406 individual proteins were identified. Among all of the identified proteins between the Kerra™ extract and control (0.1% DMSO), a total of 2196 (64%) and 1210 (36%) proteins were identified in high confidence (≤1% FDR, ≥2 unique peptides) and low confidence (≥1% and ≤5% FDR, ≤2 unique peptides), respectively (Figure 3) (Supplementary Data S1). 

### 3.4. The Effect of Kerra™ Extract on Pathway Signaling in HCT116 Cells

**Figure 4:**
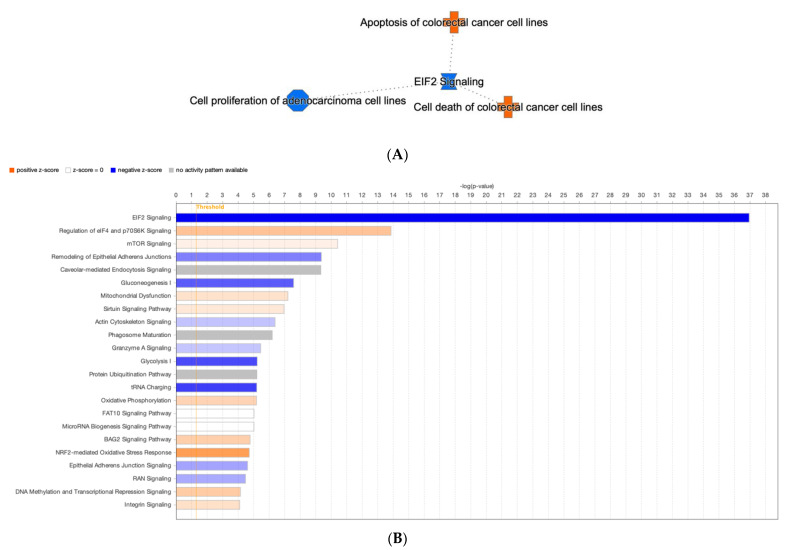
The IPA analysis revealed the identification of canonical pathways. (**A**) A network containing a compilation of the most significant results derived from IPA analysis (**B**) The threshold levels are indicated by the horizontal line. A negative z-score indicates pathway inhibition, while a positive z-score indicates pathway activation. White (transparent) bars signify “no activity” within the pathway.

A pathway signaling analysis of the entire proteome dataset of the Kerra™ extract using an IPA revealed that various pathways were activated and inhibited. The analysis revealed that the Kerra™ extract stimulated apoptosis and cell death in colorectal cancer cell lines and suppressed the cell proliferation of adenocarcinoma cell lines via the EIF2 signaling pathway (Figure 4A).

For the conical pathway signaling analysis, the IPA revealed nine significantly deactivated (z ≤ –1.5 and −log(*p*-value) > 4) and nine significantly activated (*z* ≥ 1.5, −log(*p*-value) > 4) pathways (Figure 4B). In terms of the prediction of upstream regulatory pathways in response to the Kerra™ extract in HCT116 cells, there were 13 upstream regulators (Table 1). Among these regulators, cyclin dependent kinase inhibitor 1A (CDKN1A) and MYC proto-oncogene, bHLH transcription factor (MYC) exhibited the highest and lowest activation *z*-scores, respectively. 

### 3.5. Apoptosis Protein Level Quantification

**Figure 5:**
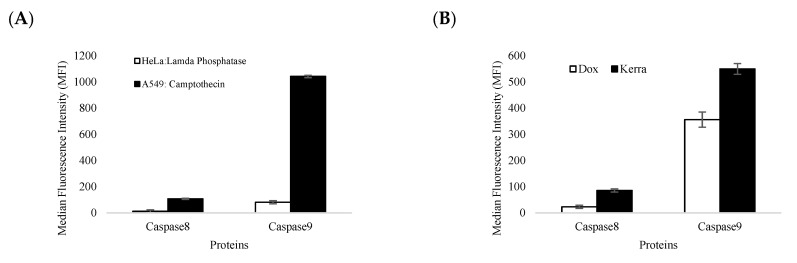
The levels of caspase-8 and caspase-9 expression were determined. (**A**) The efficiency and accuracy of immune-based reactions with (black bar) and without apoptotic stimulant compound (white bar) in A549 and HeLa reference cell lines. (**B**) The effect of Kerra™ extract on the levels of caspase-8 and caspase-9 in HCT116 cells was measured. The Dox treatment group is represented by white bars, while the Kerra™ treatment group is represented by black bars. The error bars indicate ± S.D.

For confirmation of the apoptotic-related protein expression of the Kerra™-extract-induced apoptotic event, we used the immuno-based Luminex^®^ assay, which allows the simultaneous detection of two apoptotic-related proteins that are markers of the apoptotic signaling pathways. These were caspase-8 (Asp384) and caspase-9 (Asp315). We used HeLa cells treated with lamda phosphatase as the negative control (unstimulated cells) and A549 cells treated with 5 µM of camptothecin as the positive control (apoptotic cells). The results showed that the all-apoptotic marker proteins in the positive control were in significantly higher abundance than the negative control by more than 100-fold (Figure 5A). These findings confirm that the immuno-based Luminex^®^ assay can be used to quantify the apoptotic proteins in our experiment. 

The efficiency and accuracy of immune-based reactions were assessed. It was observed that camptothecin significantly increased the levels of apoptotic marker proteins, including caspase-8 and caspase-9, by more than 10-fold compared to lambda phosphatase. These findings provide strong evidence that the immune-based assay used to quantify caspase-8 and caspase-9 was reliable and valid. The expression of apoptotic protein markers was significantly elevated by the Kerra™ extract, with caspase-8 and caspase-9 exhibiting increases of more than 3.7-fold and 1.5-fold, respectively, compared to Dox (Figure 5B).

## 4. Discussion

Interestingly, the Kerra™ extract exhibited a higher induction of late apoptosis and cell death in HCT116 cells compared to Dox at a concentration of 0.059 μM. Our results demonstrated that under Dox conditions, the percentage of HCT116 cells exhibiting total apoptosis characteristics was 20.65% (Figure 2B). Furthermore, the apoptosis levels in HCT116 cells could be further increased by raising the Dox concentration to 1 or 10 μM [32]. Additionally, prolonged treatment with Dox also resulted in late apoptosis and cell death in HCT116 and MCF7 cells after an incubation period of 5 days [33]. Therefore, the observed apoptotic characteristics in these cells imply that certain phytochemical compounds present in the Kerra™ extract have the ability to induce late apoptosis and cell death in HCT116 cells more effectively than Doxorubicin alone, specifically at this dosage. Various studies have revealed that the Dox treatment in HCT116 cells is effective in a range from 50 nM to 1.5 μM [34,35,36]. Dox is an anti-cancer drug widely used in chemotherapy for the treatment of various cancers [37]. It exerts its therapeutic effects by inducing various biological cellular changes, including cellular apoptosis. The effectiveness of Dox depends on both the dosage administered and the types of cells it targets [38,39]. This represents a research gap that needs to be addressed in future studies, aiming to identify the specific phytochemicals or combinations of phytochemicals responsible for inducing cell apoptosis. For confirmation that apoptotic HCT116 cells can be induced by the Kerra™ extract, we performed the Kerra™ extract treatment again with a 2-fold higher concentration (146 μg/mL): the percentage of apoptotic cells dramatically increased. These findings suggest that the Kerra™ extract has the ability to induce cell apoptosis in HCT116 cells in a dose-dependent manner. However, the biochemical mechanisms underlying this effect are not yet fully understood, but the results of the study suggest that the Kerra™ extract has potential as a therapeutic agent for colon cancer.

To gain a better understanding of the effect of Kerra™ on cellular responses, studying the interaction between proteins is crucial for understanding their overall biological significance. Many proteins require interactions with specific partners to function properly. Therefore, analyzing the relationships between differentially expressed proteins is valuable in gaining insights into the integral biological roles of these proteins. To achieve this, we utilized the IPA tool to conduct a network analysis, using microarray data from published literature as the basis for our investigation [40]. All the differentially expressed proteins were shown to be involved in 18 conical pathway networks. Based on the *z*-scores and *p*-values, the EIF2 signaling (*p*-value = 1.13 × 10^−37^) was most affected by the Kerra™ extract treatment in the cells. 

**Figure 6:**
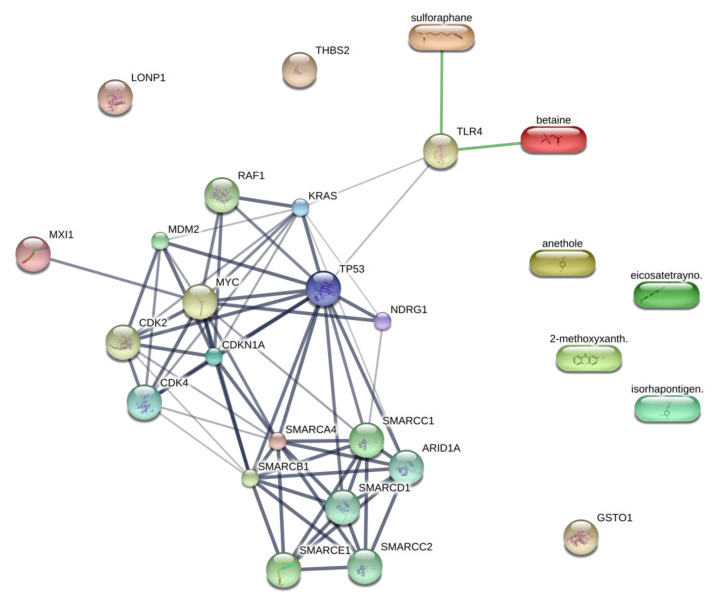
Protein upstream regulators and phytochemicals in Kerra™ extract interaction prediction. Ligand protein mapping was constructed from 2-methoxy-xanthen-9-one, isorhapontigenin, betaine, anethole, and eicosatetraynoic acid with the upstream regulators. The predicted interactions are shown in the connecting lines.

The current understanding of the upstream regulators influencing the Kerra™ extract on HCT116 cells remains limited. This study aims to enhance our knowledge of the molecular function of the Kerra™ extract and its upstream regulators. The report emphasizes key protein regulators, such as CDKN1A and MYC. Previous studies have demonstrated that Curcumin can induce apoptosis by causing G1 cell cycle arrest in a human adenocarcinoma cell line, independently of TP53, while also simultaneously inducing CDKN1A expression [41]. Our findings revealed potential upregulators as protein candidates for further investigation in relation to the development of the Kerra™ extract for colorectal cancer therapeutic approaches. In order to identify promising protein upstream regulators, we utilized previously obtained phytochemical data from the Kerra™ extract [7]. We focused on the five most abundant phytochemicals: 2-methoxy-9H-xanthen-9-one, iso-rhapontigenin, betaine, anethole, and eicosatetraynoic acid. To explore the interactions between these phytochemicals and upstream proteins, we employed the STITCH protein–ligand interaction tool (accessed on 21 May 2023) [42]. We discovered that betaine has a direct interaction with TLR4 (score = 0.82), which is one of the candidate protein upstream regulators (Figure 6). However, the remaining four phytochemicals did not exhibit any interaction with the protein upstream regulators. 

Our results showed that TRL4 also interacts with KRAS and TP53, both of which are upstream candidate proteins identified in our results. We aimed to investigate the potential of integrating proteomics data, pathway analysis, and phytochemicals from the Kerra™ extract to study chemical compounds within biological pathways. Consequently, these findings serve to support our research and provide guidance for understanding the biochemical mechanisms of Kerra™ in HCT116 cells. It is suggested that the Kerra™ extract could potentially induce apoptotic events in HCT116 cells through TLR4. 

Apoptosis, a fundamental physiological process, plays a critical role in the normal development and maintenance of multicellular organisms. In humans, the cells exhibit two primary apoptotic pathways: the intrinsic pathway, which involves caspase-9 activation and is triggered by mitochondrial dysfunction, and the extrinsic pathway, which involves caspase-8 activation and is initiated by the activation of cell surface receptors [43,44]. An immuno-based protein analysis revealed that both caspase-8 and caspase-9 were overexpressed under the Kerra™ treatment condition compared to Dox. Furthermore, the expression pattern of these proteins correlated with characteristic biochemical changes associated with apoptosis (Figure 2). Based on these findings, we can infer that the Kerra™ extract may induce apoptosis in HCT116 cells through the regulation of caspase-8 and caspase-9.

## 5. Conclusions

The Kerra™ extract from an ancient Thai scripture affected HCT116 cell viability. In addition, the extract also induced apoptosis in the cells. The Kerra™ extract affected various cellular proteins and biochemical pathways. A proteomics analysis revealed that 3406 proteins were affected by the Kerra™ extract. Using a pathway analysis, we found that the Kerra™ extract can activate apoptosis and cell death in colorectal cancer cell lines and suppress cell proliferation of adenocarcinoma cell lines via the EIF2 signaling pathway. CDKN1A and MYC were predicted as upstream regulators in response to the Kerra™ extract in the cells. Therefore, this study provides evidence for the ability of natural extracts to induce apoptosis in HCT116 colon cancer cells, demonstrating their potential as therapeutic agents for this type of cancer. Further research is needed to fully understand the mechanisms underlying these effects and to develop safe and effective therapies.

## Figures and Tables

**Table 1:** Upstream protein regulators predicted to be activated (positive value of activation *z*-score) or inhibited (minus value of activation *z*-score) in HCT116 cells after Kerra™ extract treatment.

Upstream Regulator	Molecular Function	Activation *z*-Score	*p*-Value
MYC	Transcription regulator	−1.83	1.19 × 10^−3^
HIFA	Transcription regulator	−1.75	1.45 × 10^−1^
LONP1	peptidase	−1.23	1.2 × 10^−8^
TLR4	Transmembrane receptor	−1	5 × 10^−3^
THBS2	-	−1	1.26 × 10^−3^
KRAS	Enzyme	−0.29	1.4 × 10^−2^
SFN	-	−0.25	9.18 × 10^−3^
SMARCA4	Transcription regulator	0	4.4 × 10^−2^
NDRG1	Kinase	0.17	1.27 × 10^−2^
TP53	Transcription regulator	0.78	3 × 10^−4^
MXI1	Transcription regulator	1	8.3 × 10^−3^
GSTO1	Enzyme	1.99	5.77 × 10^−1^
CDKN1A	Kinase	2.73	8.95 × 10^−2^

## Data Availability

Upon reasonable request, the corresponding author is willing to provide the data and materials supporting the results of this study.

## Supplementary Materials

The following supporting information can be downloaded at: https://www.mdpi.com/article/10.3390/medicina59081376/s1. Table S1: Protein expression data.
